# Quantitative and qualitative tremor evaluation after MR-guided focused ultrasound thalamotomy

**DOI:** 10.3389/fneur.2025.1594382

**Published:** 2025-05-02

**Authors:** Veronika Purrer, Tara Chand, Emily Pohl, Hannah Weiland, Valeri Borger, Carsten Schmeel, Henning Boecker, Ullrich Wüllner

**Affiliations:** ^1^Department of Neurology, University Hospital Bonn, Bonn, Germany; ^2^German Center of Neurodegenerative Diseases (DZNE), Bonn, Germany; ^3^Jindal Institute of Behavioural Sciences, O. P. Jindal Global University, Sonipat, India; ^4^Department of Psychiatry and Psychotherapy, Jena University Hospital, Jena, Germany; ^5^Department of Clinical Psychology, Friedrich Schiller University Jena, Jena, Germany; ^6^Department of Diagnostic and Interventional Radiology, University Hospital Bonn, Bonn, Germany; ^7^Department of Neurosurgery, University Hospital Bonn, Bonn, Germany; ^8^Department of Neuroradiology, University Hospital Bonn, Bonn, Germany

**Keywords:** tremor, thalamotomy, MRgFUS, accelerometry, quantitative measurements

## Abstract

**Introduction:**

Tremor syndromes are common neurological disorders, usually distinguished by clinical examination. Ordinal rating scales are widely used to rate tremor severity but are limited by subjective observation, interrater reliability, ceiling effects and lack of knowledge about sensitivity to change emphasizing the relevance of quantitative methods.

**Methods:**

To assess tremor characteristics in essential tremor (ET) and Parkinson’s disease tremor (PT) quantitatively, we used a wearable triaxial accelerometer in comparison to a common clinical rating scale. Furthermore, different activation conditions and changes after treatment with MR-guided focused ultrasound (MRgFUS) were examined concomitantly. Patients with disabling, medication-refractory ET (*n* = 35) or PT (*n* = 21) undergoing unilateral MRgFUS thalamotomy were assessed before, 1, 6 and 12 months after MRgFUS treatment. Clinical assessments included the Clinical Rating Scale for Tremor (CRST) and accelerometric recordings at rest, posture and kinetic movement. Peak frequencies (fp), frequency width at half maximum (FWHM), tremor stability index (TSI), and half-width power (HWP) were extracted from the power spectrum of acceleration and compared to the CRST.

**Results:**

We observed moderate to strong correlations between CRST subscores and log-transformed HWP, whereas significant correlations were only evident in ET when groups were evaluated separately. Fp, FWHM and TSI showed no differences between groups and conditions. Further, repeated measurements after MRgFUS treatment revealed significant changes of tremor severity in both, clinical rating and accelerometric recordings.

**Discussion:**

Tremor assessment using accelerometric recordings provided a fast and investigator independent method for tremor characterization and quantitative assessment, which were sensitive to changes after therapeutic interventions.

## 1 Introduction

Tremor is a common neurological symptom and defined as an involuntary, rhythmic movement. According to the International Parkinson and Movement Disorder Society (IPMDS) tremor syndromes can be classified based on clinical and etiological features. A frequently used characteristic is the activation condition, such as rest and action tremor with the latter further subdivided into postural and kinetic tremor (1).

Essential tremor (ET) is one of the most common movement disorders and characterized by a 4-12-Hz bilateral postural and kinetic tremor of the upper limbs. Other parts of the body may also be affected and, particularly with long disease duration, accompanying rest tremor may occur (2–4).

In contrast, the typical tremor in Parkinson’s disease (PD) is an asymmetric 4-6-Hz tremor at rest, which occurs in 75% of patients at the beginning or during the course of the disease. In some cases, an additional, less pronounced action tremor may be present, which often shows the same frequency pattern.

Tremor assessment and diagnosis is mainly based on clinical characteristics and may be challenging particularly in advanced stages with overlapping clinical phenotypes. For example, a previous study reported false diagnoses in about 1 in 3 ET patients, with PD being the most common false diagnosis (5, 6). In addition, 2 in 10 patients with PD receive misdiagnosis and the rate could be even higher in tremor-dominant PD (7). As misdiagnosis may result in suboptimal treatment or incorrect prognosis, a careful tremor examination is crucial.

Clinical rating scales are widely used both in clinical applications and in research studies to assess tremor severity. Common rating scales such as the Clinical Rating Scale for Tremor (CRST) developed by Fahn, Tolosa and Marin (8), the Bain and Findley Clinical Tremor Rating Scale (9) or the Tremor Research Group Essential Tremor Rating Assessment Scale (10) show overall good psychometric properties and are recommended by the IPMDS. Yet these scales require trainings to achieve good results, lack test–retest reliability, show ceiling effects in advanced tremor and lack a comprehensive analysis of sensitivity to change (11).

Therefore, quantitative methods, e.g., devices using accelerometers (“wearables”), may provide enhanced abilities to identify even small changes in tremor characteristics and may minimize variations between examiners. Indeed, electrophysiological tests can contribute to clinical characterization and diagnosis of tremor syndromes and, therefore, have been included in the axis 1 classification of tremor of the revised consensus statement of the IPMDS (1). Most commonly, tremor frequency and amplitude are a0ssessed. In addition, frequency analysis such as frequency bandwidth or cycle-to-cycle frequency variability may help characterize the rhythmicity and regularity of the tremor. Another distinction refers to the origin of the tremor, whether it either originates from a central network or mechanical-reflex oscillation. Analysis of frequency changes after weight loading addresses this differentiation as central neurogenic tremors are independent of joint inertial mass, stiffness, and reflex arc length (12). Alongside the determination of tremor frequency, these parameters have also been proposed for the characterization and differentiation of tremor syndromes (12). Further, tremor power is a reliable measure of tremor severity. The quantitative rating may especially be useful in the assessment of tremor progression or treatment response, e.g., after deep brain stimulation or Magnetic Resonance-guided Focused Ultrasound (MRgFUS) thalamotomy (13).

**Figure 1:**
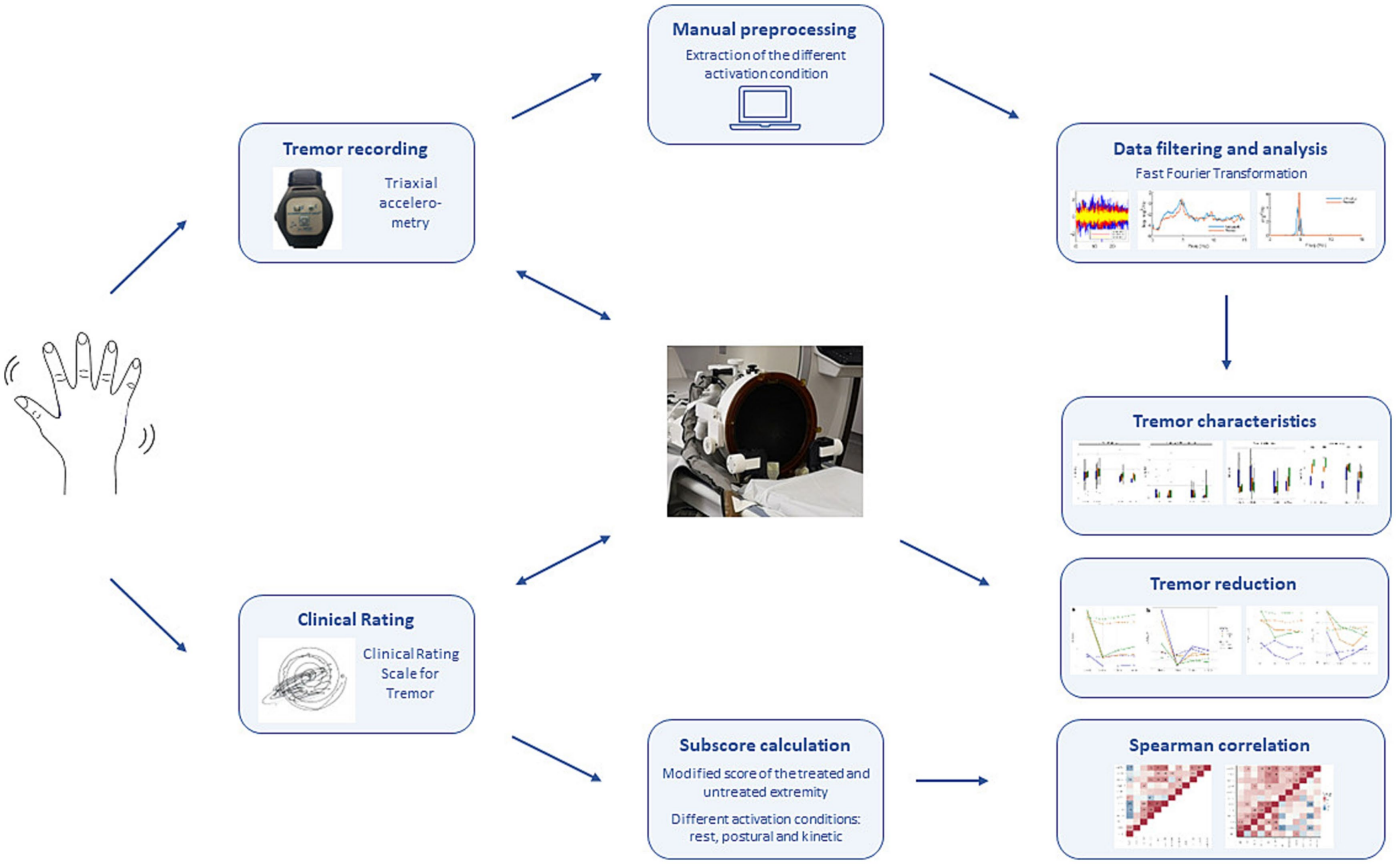
Study workflow. Data acquisition included clinical rating using the Clinical Rating Scale for Tremor (CRST) and triaxial accelerometry before and after MRI-guided focused ultrasound (MRgFUS) thalamotomy. Subscores for both extremities were calculated for the rest, postural and kinetic condition. In addition, accelerometric tremor measurements were extracted after preprocessing and filtering the raw data. Tremor characteristics in patients with essential tremor (ET) and Parkinson’s disease (PD), as well as correlations with the clinical rating and tremor outcome after MRgFUS were analyzed.

Thus, the aim of the study was to assess quantitative and qualitative outcome measures and their respective efficacy in tremor assessment using a triaxial accelerometer in clinical practice. Using a standardized assessment protocol, we also analyzed differences in tremor characteristics in ET and Parkinson’s tremor (PT). Furthermore, we explored the potential of quantitative measurements in repeated measures evaluating the treatment response after unilateral MRgFUS thalamotomy in patients with ET and PT (Figure 1).

## 2 Patients and methods

### 2.1 Patients and procedure

35 patients with disabling, medication-refractory ET and 21 with PT (reports of at least two insufficient previous medication trials) were enrolled between April 2019 and March 2023 for unilateral MRgFUS thalamotomy. The diagnosis of ET or PD was confirmed in our outpatient department by neurologists specialized in movement disorders (UW and VP, 30 and 6 years of experience in movement disorders) according to the IPMDS consensus criteria (1). A moderate to severe tremor (score of ≥ 2 in the dominant hand on the CRST) and disability in daily activities and/or quality of life (score > 2 in the disability suspicion of the CRST or ≥ 30% self-rated reduction of quality of life caused by the tremor) were required. Current medications had to been stable for at least 30 days at the time point of enrollment and were discontinued prior to treatment (1 week prior in ET and at least 12 h overnight in PD) to get the most visible tremor. Exclusion criteria involved structural brain damage, epilepsy, coagulopathies, severe cardiac conditions, history of psychiatric disorders or substance abuse, reported cognitive impairment or a skull density ratio < 0.3. MRgFUS treatment was performed following the established treatment protocol, which has been described previously (14, 15). The study was performed according to the Declaration of Helsinki and approved by the local Ethics Committee (314/18). All participants provided written, informed consent.

### 2.2 Clinical evaluation

Clinical evaluation was conducted by a trained neurologist (UW or VP) directly before treatment (T0) as well as 1 to 3 days after treatment (T1). Follow-up visits were conducted 6 months (T2) and 12 months (T3) after MRgFUS (Supplementary Figure S1a). The raters were not blinded to the patient’s or time points of follow-up. To avoid levodopa-induced modification of tremor amplitude, tremor assessment in PD patients was performed in “OFF” condition after at least 4 h of medication withdrawal.

#### 2.2.1 Qualitative tremor assessment

The CRST was used for qualitative tremor assessment; raters were blinded to the accelerometry results. To compare the tremor improvement of the treated hand (= contralateral to the thalamotomy side), we used a hand-specific subscore combining part A and B of the treated upper extremity (CRST_mod_, details provided in Supplementary Methods S1). To compare the different activation conditions (rest, postural and kinetic tremor), the corresponding items of the clinical observation (Part A) were obtained for both hands (treated and untreated extremity) and each condition (rest, postural, kinetic) separately (each score ranging from 0 to 4). Higher values indicate more severe tremor.

#### 2.2.2 Quantitative tremor assessment

Quantitative tremor assessment was conducted with a CE-approved triaxial accelerometry (SOMNOwatch™ plus®, SOMNOmedics, Randersacker, Germany). For time-matched comparisons, qualitative and quantitative tremor assessments were obtained subsequently on the same day. Data acquisition and analysis were performed by different investigators.

The device was placed on each side on the proximal one-third of the metacarpus (Supplementary Figure S1b). While the patient was seated comfortably in an armchair, tremor recordings were obtained bilaterally in rest (R) and forward outstretched postural condition (without (P) and with weight loading (PW) using a 1,000 mL water-filled bottle) for 30 s each as well as kinetic (K) (finger-to-nose maneuver) condition for 15 s (Supplementary Figure S1c). Using the proprietary software (DOMINOlight; SOMNOmedics), the first and last 3–5 s of each recording (based on clinical observation during the recording) were removed to avoid measurements of arbitrary movements for initiating or terminating the exercise or distortions of the power spectrum caused by the short-term arrest in re-emergent tremor and raw data was downloaded with the software.

Recordings of accelerometric signals were conducted with 128 Hz. Data processing was performed using Matlab (MathWorks, Inc., USA, R2023b). To determine the normalized power distribution of the tremor in the frequency band 1 to 20 Hz, Fast Fourier Transform (FFT) analysis was used. The following spectral parameters were extracted and means were calculated for the eight conditions: peak frequency (f_p_, Hz; frequency with maximum power in the power spectrum within the range of 2–15 Hz), frequency width at half maximum (FWHM, Hz; a measure of the frequency variability within the entire signal) (16), tremor stability index (TSI, Hz; a parameter of stability of tremor frequency over time) (17), and half-width power (HWP, mg; a measure of tremor power under the main spectral frequency peak between the frequency range of 2–15 Hz) (18). As the algorithm calculates values for all subjects even if there is no veritable oscillatory component, subjects without obvious tremor peak in the power spectrum of the respective activation condition were excluded from the analysis of f_p_, FWHM and TSI (Supplementary Figure S5). Details of tremor analysis are provided in the Supplementary Methods S2, Supplementary Figures S2–S7.

### 2.3 Statistical analysis

Statistics were performed using RStudio (2023.12.0 + 369, R Foundation for Statistical Computing, Vienna, Austria). Evaluation of the normal distribution of the data was performed using the Shapiro–Wilk test. Group differences in demographics and CRST scores were measured with the Fisher’s exact test or Wilcoxon signed-rank test. To control for multiple comparisons and address the large number of relationships tested, we applied the false discovery rate (FDR) correction method. Unlike the Bonferroni method, which is more conservative and adjusts the significance threshold equally across all comparisons, the FDR method is less stringent. It is better suited for situations where variables are not completely independent, thereby reducing the risk of a Type II error (19).

Correlations between qualitative (CRST) and quantitative tremor measurements (log-transformed HWP) were assessed using Spearman’s correlation coefficient *r_s_*.

To assess differences in accelerometric tremor characteristics in different activation conditions among the groups, a linear mixed model with group (ET and PD), condition (R, P, K) and their interaction as fixed effects was used. Model assumptions (linearity, normality, homoscedasticity, and independence of residuals) were assessed visually using residuals vs. fitted plots and Q-Q plots. All diagnostic checks supported the validity of the model. In case of violations of homoscedasticity, the model was adjusted to account for unequal variances. A simple *t*-test and FDR correction were applied for post-hoc pairwise tests and adjustment for multiple comparisons. Not normally distributed variables (f_p_, TSI, FWHM, HWP) were log transformed prior to analysis.

To identify the origin of the oscillator, changes of tremor frequencies in postural condition with and without weight loading were calculated. Changes <1 Hz supposedly indicate central tremor (20).

The Friedman’s test, with pairwise comparison post-hoc tests and FDR corrections, was used to assess significant within-group changes of the tremor scores and accelerometric measurements among all time points. Effect sizes *r* were calculated using the following formula for nonparametric data in which *Z* is the test statistic and *n* is the number of observations (21): $r=|\frac{Z}{\sqrt{n}}|$ . An effect size *r* < 0.3 is considered a small effect, 0.3 < *r* < 0.5 a medium effect and *r* > 0.5 a large effect. *p*-values <0.05 were considered statistically significant. Data are presented as mean ± standard deviation.

## 3 Results

### 3.1 Demographical and clinical characteristics

Characteristics of ET and PT patients are provided in Table 1. Mean age, age of onset, disease duration and tremor scores at baseline significantly differ between groups. 31 (89%) ET and 13 (62%) PT patients underwent left sided thalamotomy (*p* = 0.040). At baseline, a significant asymmetry between the treated and untreated extremity was present in PT (*p* < 0.001). Rest tremor subscores of the treated extremity were significantly higher in PT patients, while postural and kinetic tremor subscores were higher in ET, but only reached significance for the kinetic scores. ET patients reached significantly higher values for the postural and kinetic condition of the untreated extremity than PT patients.

**Table 1:** Demographic and clinical characteristics of the study participants (*n* = 56).

Characteristic	ET (*n* = 35)	PD (*n* = 21)	*p*-value^†^
Age – yr*	70.5 ± 12.9	62.6 ± 10.8	0.004
Male sex – no. (%)	27 (77%)	17 (81%)	1.0
Right-handedness – no. (%)	33 (94%)	19 (90%)	0.626
Age of onset*	39.5 ± 22.0	54.7 ± 14.2	0.019
Disease duration*	31.0 ± 18.5	8.0 ± 11.4	<0.001
CRST at baseline*			
Total score^#^	59.8 ± 17.1	31.0 ± 15.0	59.8 ± 17.1
Treated arm (CRST_mod_)^‡^	19.1 ± 4.9	13.8 ± 5.5	19.1 ± 4.9
Untreated arm (CRST_mod_)^‡^	16.9 ± 5.6	6.0 ± 5.3	16.9 ± 5.6
Rest, treated arm (CRST_R_)^ǁ^	0.7 ± 0.9	3.4 ± 0.5	0.7 ± 0.9
Rest, untreated arm (CRST_R_)^ǁ^	0.5 ± 0.7	0.9 ± 0.9	0.5 ± 0.7
Postural, treated arm (CRST_P_)^ǁ^	3.1 ± 0.8	2.7 ± 1.1	3.1 ± 0.8
Postural, untreated arm (CRST_P_)^ǁ^	2.7 ± 0.9	1.0 ± 1.1	2.7 ± 0.9
Kinetic, treated arm (CRST_K_)^ǁ^	3.2 ± 0.9	1.4 ± 1.2	3.2 ± 0.9
Kinetic, untreated arm (CRST_K_)^ǁ^	2.8 ± 1.1	0.6 ± 0.9	2.8 ± 1.1

*Values are means ± SD.

^#^Consisting of subscores A (clinical observation), B (motor tasks) and C (subjective disability). The total score ranges from 0 to 144.

^‡Treated = modified score of the clinical examination and motor tasks of the treated upper extremity contralateral to the treated cerebral hemisphere, untreated = modified score of the clinical examination and motor tasks of the untreated upper extremity ipsilateral to the treated cerebral hemisphere. Subscores ranges from 0 to 28 each.^

^ǁ^Item for rest, postural or kinetic tremor in the treated or untreated upper limb (Part A); each item ranges from 0 to 4.

^†^Group differences were calculated using Fisher’s exact test and the Wilcoxon signed-rank test. *p*-values were adjusted using the False Discovery Rate (FDR) method to control the significance level across all performed tests. A *p*-value < 0.05 was considered statistically significant.

ET, Essential Tremor; PD, Parkinson’s Disease; CRST, Clinical Rating Scale for Tremor; CRST_R_, subitem for rest tremor; CRST_P_, subitem for postural tremor; CRST_K_, subitem for kinetic tremor.

#### 3.1.1 Quantitative tremor characteristics

When comparing the subscores of the CRST with quantitative tremor power (log-transformed HWP), we overall observed moderate correlations for postural tremors of both extremities (treated: rs = 0.35, *p* = 0.010; untreated: rs = 0.48, *p* < 0.001) and rest (rs = 0.35, *p* = 0.039) and kinetic (rs = 0.38, *p* = 0.002) tremor of the treated extremity as well as strong correlation for kinetic tremors of the untreated extremity (rs = 0.68, *p* < 0.001).

**Figure 2:**
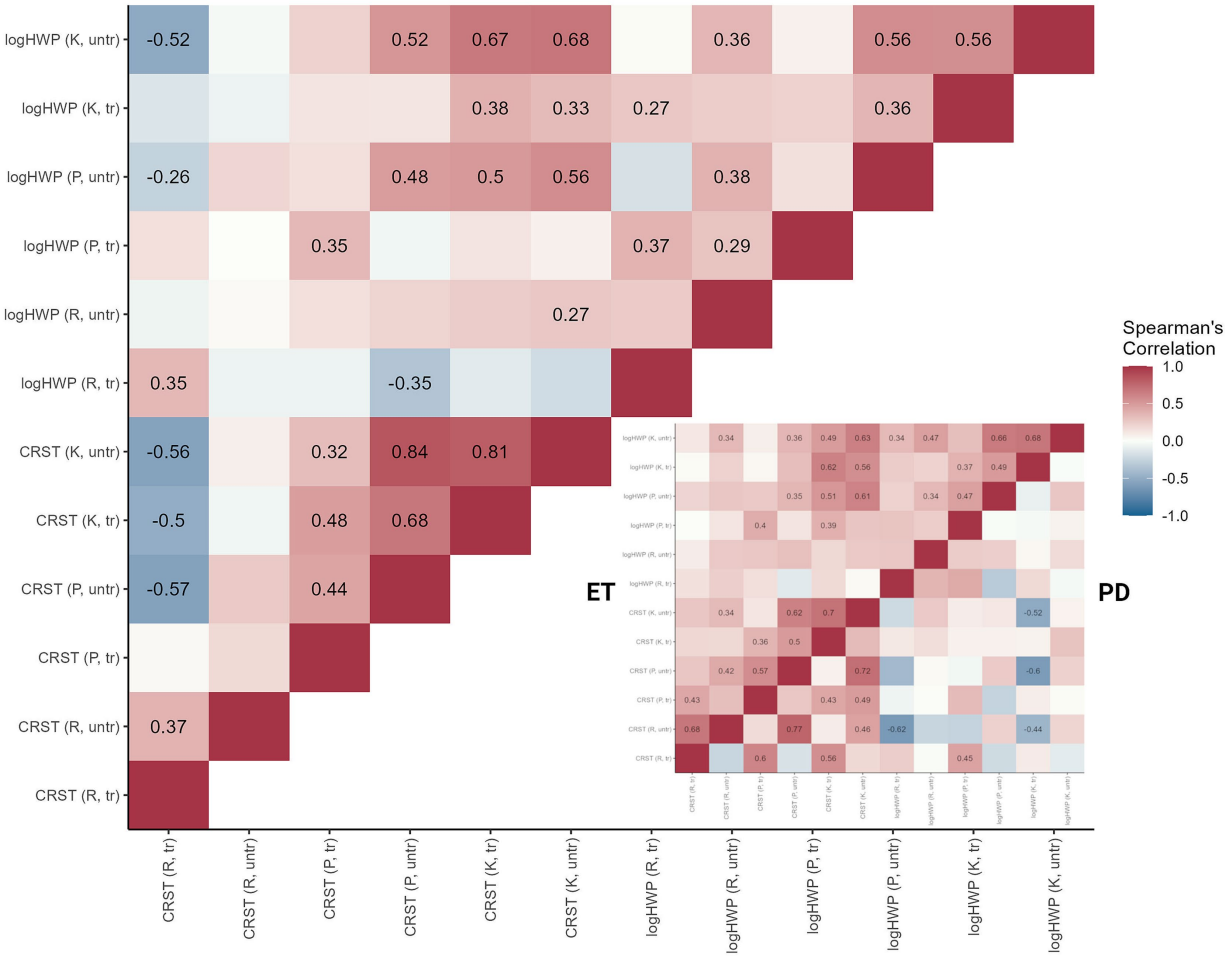
Spearman correlation statistics (rho) for the relationship between clinical rating (Clinical Rating Scale for Tremor (CRST)) and tremor power (half width power (HWP)). The larger half square (*left*) displays the correlations for all study participants, the small square (*right*) the correlations for ET and PD patients separately. Spearman’s correlation coefficients *r_s_* are given for significant correlations. Shades of red indicate a positive, shades of blue a negative association.

Comparing ET and PD patients separately, significant correlations could only be observed in ET patients (postural, treated: rs = 0.40, *p* = 0.016, postural, untreated: rs = 0.35, *p* = 0.037, kinetic, treated: rs = 0.62, *p* < 0.001, kinetic, untreated: rs = 0.63, *p* < 0.001) (Figure 2).

**Figure 3:**
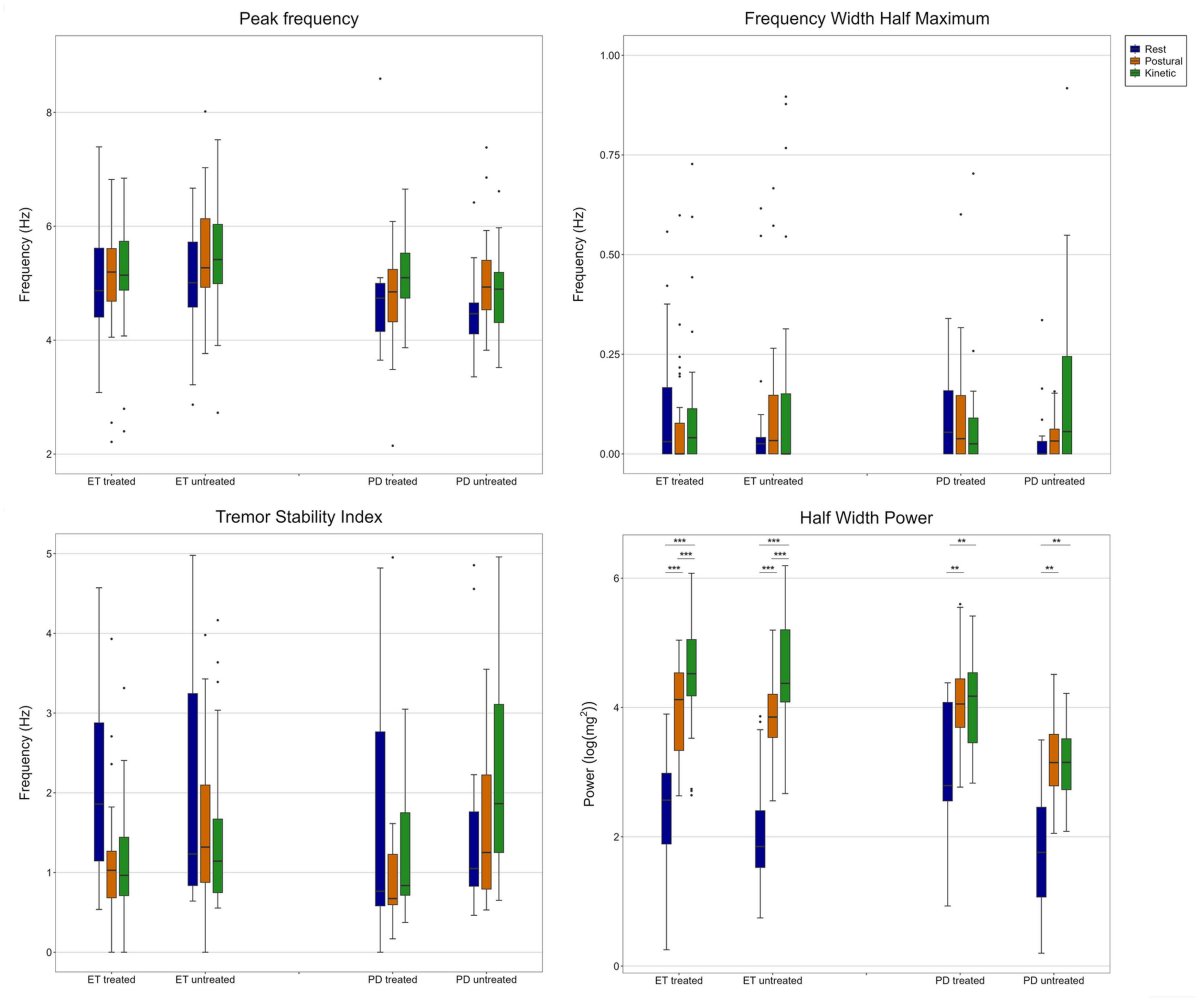
Quantitative tremor characteristics at baseline. Peak frequency (f_p_), frequency width half maximum (FWHM), tremor stability index (TSI) and half width power (HWP) are presented for each condition [rest (*blue*), postural (*orange*) and kinetic (*green*)] and extremity (treated and untreated) across ET and PD patients.

Tremor characteristics at baseline derived by accelerometry are shown in Figure 3.

In both, ET and PD, changes of fp in postural condition with and without weight loading were <1 Hz, indicating a central origin of oscillation (ET, treated extremity: fp(P) = 5.12 ± 1.00 vs. fp(PW) = 5.05 ± 0.96; ET, untreated extremity: fp(P) = 5.75 ± 1.70 vs. fp(PW) = 5.47 ± 1.54; PD, treated extremity: fp(P) = 4.67 ± 0.82 vs. fp(PW) = 5.23 ± 0.87; PD, untreated extremity: fp(P) = 5.07 ± 0.92 vs. fp(PW) = 5.06 ± 1.07).

There was no significant effect of group, condition or interaction (group*condition) on fp and FWHM of the treated and untreated extremity. The TSI of the untreated extremity showed a significant effect of condition and the interaction between group and condition but post-hoc pairwise comparisons revealed no significant differences. Comparing HWP among groups and conditions, a significant effect of the group was found for the treated extremity (*t*(130.9) = 2.79, *p* = 0.006) which, however, did not achieve significant differences in *post-hoc* analysis. Condition (treated: *t*(90.7) = 6.48, *p* < 0.001; untreated: *t*(84.5) = 7.84, *p* < 0.001) and interaction between group and condition (treated: *t*(91.3) = −3.19, *p* = 0.002; untreated: *t*(84.2) = −3.78, *p* < 0.001) revealed significant differences for both extremities. In ET and both extremities, *post-hoc* analysis showed significantly lower values for rest tremor compared to postural (treated: *p* < 0.001; untreated: *p* < 0.001) and kinetic (treated: *p* < 0.001; untreated: *p* < 0.001) tremor and lower values of postural tremor compared to kinetic tremor (treated: *p* = 0.001; untreated: *p* < 0.001). In PD, no differences were found comparing postural and kinetic condition, but rest tremor was significantly lower compared to postural (treated: *p* = 0.002; untreated: *p* = 0.005) and kinetic (treated: *p* = 0.006; untreated: *p* = 0.002) tremor in both extremities.

### 3.2 Clinical outcome after MRgFUS

In both, ET and PD, a significant tremor reduction of the treated extremity was observed at all follow-up time points on the modified CRST (*p* < 0.001 in both) and the subscales for rest (*p* < 0.001 in both), postural (*p* < 0.001 in both) and kinetic (*p* < 0.001 in both) tremor, with the most beneficial effect being achieved immediately after treatment.

Similarly, HWP showed significant reductions of tremor power of the treated extremity in all conditions except of rest tremor in ET patients (PD, rest: *p* = 0.023; ET, postural: *p* < 0.001; PD, postural: *p* < 0.001; ET, kinetic: *p* < 0.001; PD, kinetic: *p* = 0.005).

**Figure 4:**
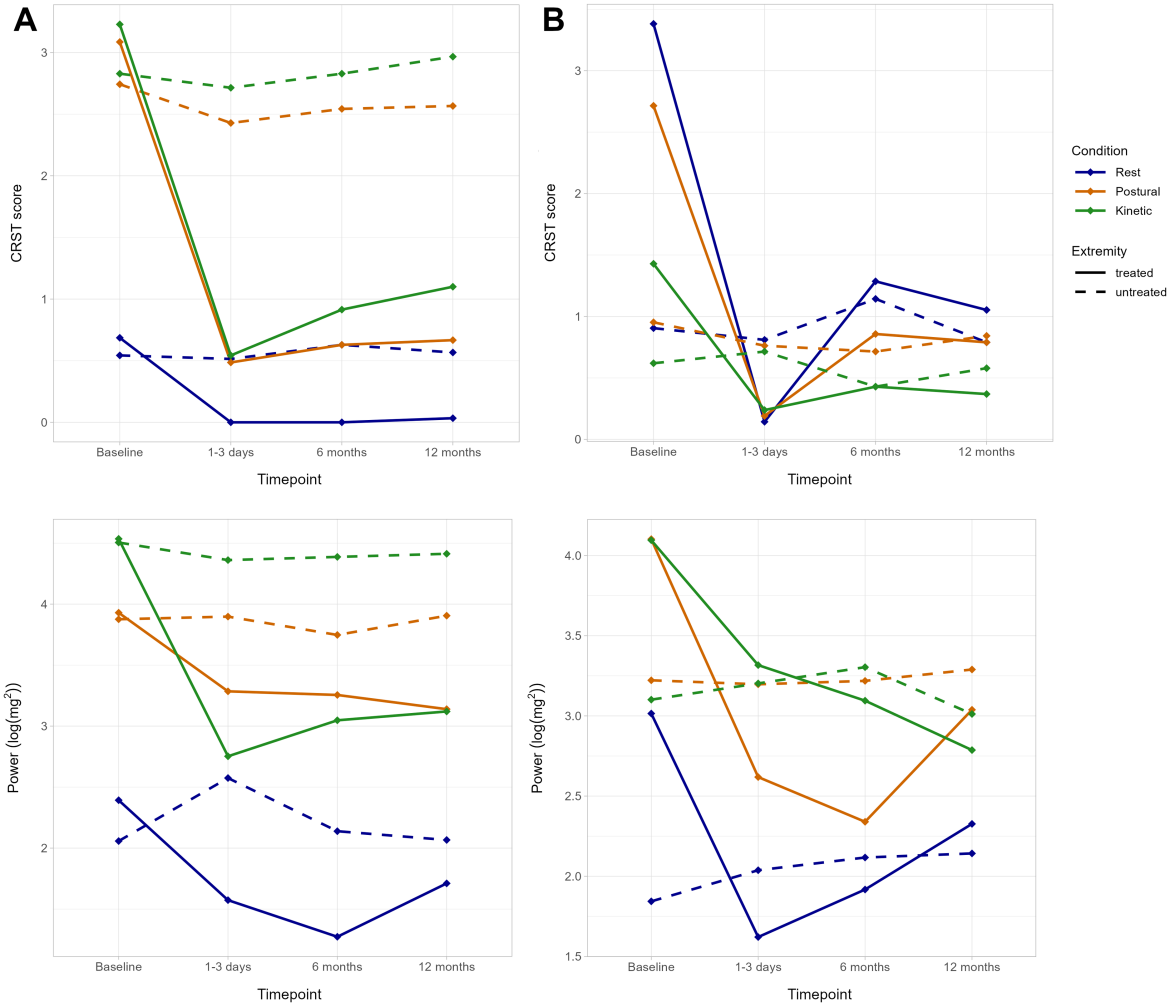
Mean tremor improvement after MRI-guided focused ultrasound (MRgFUS) thalamotomy in patients with **(A)** essential tremor (ET) and **(B)** Parkinson’s disease (PD). **(A)** Mean subscores for rest (*blue*), postural (*orange*) and kinetic (*green*) condition on the Clinical Rating Scale for Tremor (CRST) and mean tremor power (half width power) are shown for the treated (*solid line*) and untreated (*dashed line*) extremity separately.

No significant changes in CRST scores and HWP were observed for the untreated extremity (Figure 4, Supplementary Table S1). In ET, a significant reduction of fp in kinetic condition of the treated extremity was found at T1 (*r* = 0.46, *p* = 0.038). No other changes in tremor characteristics (fp, TSI, FWHM) could be observed (Supplementary Figure S8).

## 4 Discussion

Tremor syndromes often can be distinguished by patient’s history and clinical findings, such as appearance of the tremor and other neurological features. Therefore, a careful physical examination is crucial for diagnosis, focusing in particular on tremor distribution and characteristics (e.g., frequency, activation condition, amplitude) (1). Clinical rating scales can further aid to assess tremor quantitatively and monitor progression or treatment effects.

The CRST is commonly used to assess tremor symptoms and shows good psychometric properties. Nevertheless, ordinal scales are limited by ceiling effects, especially in advanced tremors, and tremor amplitudes are perceived logarithmic rather than linear by raters (11, 13). Furthermore, less is known about sensitivity to change, an effect that is particularly critical in treatment trials or longitudinal studies. Therefore, standardized measurements for direct quantification of tremor acceleration are highly recommended. Accelerometer-based devices are easy to use, cost-effective and reliable in the assessment of power spectra of acceleration and frequencies, even in longitudinal studies and between different raters (22, 23). Despite the increasing application of these devices, there is still a lack of standardized protocols and validation compared to clinical ratings. In addition, tremor evaluation in different activation conditions is crucial as tremor often varies and tremor syndromes can often be distinguished by these differences (1). Therefore, we used a standardized protocol to capture accelerometric and clinical rating in clinically well-defined patients with ET and PD and different activation conditions (rest, posture and kinetic movement). We observed moderate to strong correlations between the CRST and log-transformed HWP, which aligns with previous studies comparing clinical tremor ratings with accelerometry-based measures (13, 24, 25). Log transformation was used in accordance with the Weber-Fechner law of psychophysics, as recommended earlier (24, 26).

To characterize tremor electrophysiologically, it is proposed to investigate tremor frequency, regularity and the (presumed) origin of oscillation(s) (12). Tremor characteristics were analyzed using a linear mixed model, which accounts for within-subject variability and enhance statistical robustness. We found no differences between ET and PD patients as well as activation conditions for f_p_, FWHM and TSI. To avoid bias caused by non-oscillatory movements, we excluded measurements without an obvious peak in the power spectrum by visual inspection (12). With some exceptions, tremor frequency usually does not enable differentiation of tremor syndromes as it usually ranges from 4 to 8 Hz in pathological tremor syndromes (1). Tremor frequencies of the ET and PD patients in our study were also within this range. Moreover, changes of f_p_ after weight loading were less than 1 Hz compared to postural hold without weight, indicating a central network oscillation as known in ET and PD (1, 12). To assess tremor variability, we used the TSI and FWHM. TSI is a measure of cycle-to-cycle variability over time, thus reflecting the time-varying behavior of a single oscillator (16, 17). Whereas FWHM measures the range of frequencies within the entire power spectrum and is proposed to characterize the range of multiple oscillators within the signal (16). For both parameters, we found no differences between groups, conditions or overtime. Furthermore, FWHM was <2 Hz, which is considered as a high rhythmic pattern (12). Previous studies also failed to demonstrate differences between ET and PD patients or different activation conditions (16, 27, 28). Using a resting, postural and movement task, Luft et al. found differences in TSI only for healthy controls compared to ET and PD patients but not between the two patient groups (27). Another study compared TSI and FWHM in postural, kinetic and writing condition but found no differences within ET patients (16). A TSI cut-off of 1.05 was found to differentiate effectively between ET and PT (17). However, this investigation referred to the comparison of postural tremor in ET and rest tremor in PD, which we did not consider in this study and may explain the lack of differentiation in our cohort.

Characteristically, the ET patients showed more pronounced postural and kinetic tremors compared to rest condition. Rest tremor in ET is considered as a sign of advanced disease progression (1). Therefore, the high incidence in our cohort is not surprising, as we included patients with severe, medication-refractory ET undergoing MRgFUS treatment. PD patients showed an asymmetric tremor. Assessing tremor clinically and using accelerometric measures, controversial results were observed in PD: the CRST showed higher scores for rest tremor whereas HWP was increased in postural and kinetic condition. One possible explanation could be high variability in tremor amplitudes which have been noted in PD before (12). Although clinical ratings and tremor recordings were conducted simultaneously to avoid fluctuations over time, CRST ratings were based on the overall impression during the examination, whereas accelerometry captures only a small fraction during the 30-s recording.

To further evaluate accelerometric measurements in clinical practice, we assessed repeated tremor recordings to monitor treatment response after unilateral MRgFUS thalamotomy. MRgFUS is an emerging technique for treatment of severe tremor symptoms. To date, several studies have shown its efficacy in patients with ET and PD, mostly using clinical rating scales for tremor assessment (29–32). In the past, we also demonstrated a beneficial tremor outcome after MRgFUS in patients with ET and PD using the CRST (15, 33). To our knowledge, no previous study has used quantitative measurements to evaluate tremor outcome in MRgFUS. Only one study, evaluating a standardized accelerometric protocol in tremor patients, reported a significant and stable reduction of tremor power in a single patient with severe ET after unilateral MRgFUS thalamotomy (34). The potential of accelerometric devices in tremor recording and correlations with clinical ratings on the other hand have been demonstrated in studies of deep brain stimulation (25, 35–38). Using a triaxial accelerometer, we found a significant decrease in tremor power after MRgFUS thalamotomy in ET and PD patients. This tremor reduction was evident in almost all activation conditions of tremor (HWP_R_ did not reach significance in ET) and even 12 months after the treatment - indicating a good sensitivity to change. Consistent with previous findings, tremor reduction was most noticeable immediately after the treatment (14, 15, 29, 30, 33). Tremor measurements (HPW) achieved by accelerometry significantly correlated with clinical ratings using the CRST.

Despite evaluation of treatment efficacy in the long-term, another potential approach is the use of accelerometric measurements during the treatment procedure. This may provide a more sensitive and objective method to detect tremor changes and may optimize target verification. Given the fact that the electromagnetic MR environment can affect the accelerometric signal, a set up while MRgFUS is challenging. Recently, a few studies have referred to this (39, 40). E.g., using a MR-compatible accelerometer, near real-time visualization and quantification of tremor was demonstrated in 20 MRgFUS treatments showing strong correlations with the standard clinical assessment, the CRST (39).

There are several limitations that must be mentioned. First, the sample size in each group was small. However, clinical characteristics were appropriate and tremor improvement after MRgFUS was evident, as published previously. Second, clinical rating was not blinded. Although we tried to overcome this disadvantage by raters being blinded to the accelerometric results, video-based ratings would provide more objectivity. Although we demonstrated significant correlations between tremor ratings and accelerometric data, and quantitative measurements showed sensitivity in detecting changes in tremor severity, our analysis did not address test–retest reliability. Future studies incorporating repeated measurements and independent raters are needed to enhance reliability. Unfortunately, in the outpatient setting, only a 4-h withdrawal of dopaminergic medication was feasible, which may have influenced the motor assessment in patients with PD. A longer withdrawal period would have been preferable to minimize any residual effects of levodopa on tremor scores. However, this was not achievable due to practical and logistical constraints. Specifically, many patients relied on public transportation or private vehicles to attend outpatient appointments – often traveling long distances - which required a certain level of mobility. This limitation is particularly relevant given the observed trend toward increased dopaminergic medication over time. On the other hand, it is most likely that these adjustments were made in response to overall disease progression, as patients underwent MRgFUS thalamotomy because of insufficient tremor control despite optimized medical therapy. Our sample was not homogenous in terms of disease duration and severity as we included only patients with disabling tremor seeking MRgFUS treatment. Thus, statements on, e.g., tremor characteristics in earlier stages are limited and must be considered in future studies. Last, comparisons with other studies could be limited as we did not differentiate between the less and more affected extremity rather than the treated and untreated extremity. This was mainly done to determine the effect of MRgFUS. Moreover, in most cases the treated extremity was also the more severely affected one, suggesting no major impact on our findings.

In conclusion, using a standardized accelerometric protocol, our method reliably revealed moderate to high correlations between accelerometric measurements and clinical ratings. Tremor characteristics were consistent with the diagnosis of ET and PD. Further, stable tremor improvement in rest, postural and kinetic condition could be demonstrated up to 12 months after MRgFUS thalamotomy, both by clinical and accelerometric measurements.

Devices or wearables can provide a fast, easily implemented and investigator independent tool for quantitative tremor recording and may help to better characterize and compare the motor outcome after MRgFUS or other treatment options available for movement disorders.

## Data Availability

The raw data supporting the conclusions of this article will be made available by the authors, without undue reservation.

## References

[1] Bhatia KP Bain P Bajaj N Elble RJ Hallett M Louis ED. Consensus statement on the classification of tremors. From the task force on tremor of the International Parkinson and Movement Disorder Society. Mov Disord. (2018) 33:75–87. doi: 10.1002/mds.27121, PMID: 29193359 PMC6530552

[2] Elble RJ. Diagnostic criteria for essential tremor and differential diagnosis. Neurology. (2000) 54:S2–6. PMID: 10854344

[3] Cohen O Pullman S Jurewicz E Watner D Louis ED. Rest tremor in patients with essential tremor: prevalence, clinical correlates, and electrophysiologic characteristics. Arch Neurol. (2003) 60:405–10. doi: 10.1001/archneur.60.3.405, PMID: 12633153

[4] Louis ED Hernandez N Michalec M. Prevalence and correlates of rest tremor in essential tremor: cross-sectional survey of 831 patients across four distinct cohorts. Eur J Neurol. (2015) 22:927–32. doi: 10.1111/ene.12683, PMID: 25786561 PMC4414706

[5] Jain S Lo SE Louis ED. Common misdiagnosis of a common neurological disorder: how are we misdiagnosing essential tremor? Arch Neurol. (2006) 63:1100–4. doi: 10.1001/archneur.63.8.1100, PMID: 16908735

[6] Schrag A Münchau A Bhatia KP Quinn NP Marsden CD. Essential tremor: an overdiagnosed condition? J Neurol. (2000) 247:955–9. doi: 10.1007/s004150070053, PMID: 11200689

[7] Selikhova M Kempster PA Revesz T Holton JL Lees AJ. Neuropathological findings in benign tremulous parkinsonism. Mov Disord. (2013) 28:145–52. doi: 10.1002/mds.25220, PMID: 23239469

[8] Fahn S Tolosa E Marin C. Clinical rating scale for tremor In: Jankovic J Tolosa E, editors. Parkinson’s disease and movement disorders. Baltimore, MD and Munich, Germany: Urban & Schwarzenberg (1988)

[9] Bain PG Findley LJ Atchison P Behari M Vidailhet M Gresty M. Assessing tremor severity. J Neurol Neurosurg Psychiatry. (1993) 56:868–73. doi: 10.1136/jnnp.56.8.868, PMID: 8350102 PMC1015140

[10] Elble R Comella C Fahn S Hallett M Jankovic J Juncos JL. Reliability of a new scale for essential tremor. Mov Disord. (2012) 27:1567–9. doi: 10.1002/mds.25162, PMID: 23032792 PMC4157921

[11] Elble R Bain P Forjaz MJ Haubenberger D Testa C Goetz CG. Task force report: scales for screening and evaluating tremor: critique and recommendations. Mov Disord. (2013) 28:1793–800. doi: 10.1002/mds.25648, PMID: 24038576

[12] Deuschl G Becktepe JS Dirkx M Haubenberger D Hassan A Helmich RC. The clinical and electrophysiological investigation of tremor. Clin Neurophysiol. (2022) 136:93–129. doi: 10.1016/j.clinph.2022.01.004 35149267

[13] Elble RJ Pullman SL Matsumoto JY Raethjen J Deuschl G Tintner R. Tremor amplitude is logarithmically related to 4- and 5-point tremor rating scales. Brain. (2006) 129:2660–6. doi: 10.1093/brain/awl190, PMID: 16891320

[14] Elias WJ Huss D Voss T Loomba J Khaled M Zadicario E. A pilot study of focused ultrasound thalamotomy for essential tremor. N Engl J Med. (2013) 369:640–8. doi: 10.1056/NEJMoa1300962, PMID: 23944301

[15] Purrer V Borger V Pohl E Upadhyay N Boecker H Schmeel C. Transcranial high-intensity magnetic resonance-guided focused ultrasound (tcMRgFUS) - safety and impacts on tremor severity and quality of life. Parkinsonism Relat Disord. (2022) 100:6–12. doi: 10.1016/j.parkreldis.2022.05.017, PMID: 35640415

[16] Panyakaew P Cho HJ Lee SW Wu T Hallett M. The pathophysiology of dystonic tremors and comparison with essential tremor. J Neurosci. (2020) 40:9317–26. doi: 10.1523/JNEUROSCI.1181-20.2020, PMID: 33097635 PMC7687063

[17] Di Biase L Brittain J-S Shah SA Pedrosa DJ Cagnan H Mathy A. Tremor stability index: a new tool for differential diagnosis in tremor syndromes. Brain. (2017) 140:1977–86. doi: 10.1093/brain/awx104, PMID: 28459950 PMC5493195

[18] Raethjen J Lauk M Köster B Fietzek U Friege L Timmer J. Tremor analysis in two normal cohorts. Clin Neurophysiol. (2004) 115:2151–6. doi: 10.1016/j.clinph.2004.04.006, PMID: 15294218

[19] Benjamini Y Hochberg Y. Controlling the false discovery rate: a practical and powerful approach to multiple testing. J R Stat Soc Ser B Methodol. (1995) 57:289–300. doi: 10.1111/j.2517-6161.1995.tb02031.x

[20] Deuschl G Raethjen J Lindemann M Krack P. The pathophysiology of tremor. Muscle Nerve. (2001) 24:716–35. doi: 10.1002/mus.1063, PMID: 11360255

[21] Fritz CO Morris PE Richler JJ. Effect size estimates: current use, calculations, and interpretation. J Exp Psychol Gen. (2012) 141:2–18. doi: 10.1037/a0024338, PMID: 21823805

[22] FitzGerald JJ Lu Z Jareonsettasin P Antoniades CA. Quantifying motor impairment in movement disorders. Front Neurosci. (2018) 12:202. doi: 10.3389/fnins.2018.00202, PMID: 29695949 PMC5904266

[23] Grimaldi G Manto M. Neurological tremor: sensors, signal processing and emerging applications. Sensors. (2010) 10:1399–422. doi: 10.3390/s100201399, PMID: 22205874 PMC3244020

[24] Elble RJ. Estimating change in tremor amplitude using clinical ratings: recommendations for clinical trials. Tremor Other Hyperkinet Mov. (2018) 8:600. doi: 10.5334/tohm.455 PMC6802602 31637097

[25] Obwegeser AA Uitti RJ Witte RJ Lucas JA Turk MF Wharen RE. Quantitative and qualitative outcome measures after thalamic deep brain stimulation to treat disabling tremors. Neurosurgery. (2001) 48:274–81. doi: 10.1097/00006123-200102000-00004, PMID: 11220369

[26] Haubenberger D Kalowitz D Nahab FB Toro C Ippolito D Luckenbaugh DA. Validation of digital spiral analysis as outcome parameter for clinical trials in essential tremor. Mov Disord. (2011) 26:2073–80. doi: 10.1002/mds.23808, PMID: 21714004 PMC4117681

[27] Luft F Sharifi S Mugge W Schouten AC Lo Bour J van Rootselaar A-F. A power spectral density-based method to detect tremor and tremor intermittency in movement disorders. Sensors. (2019) 19:4301. doi: 10.3390/s19194301, PMID: 31590227 PMC6806079

[28] Rajan R Anandapadmanabhan R Vishnoi A Latorre A Thirugnanasambandam N Dipani A. Essential tremor and essential tremor plus are essentially similar Electrophysiologically. Mov Disord Clin Pract. (2024) 11:136–42. doi: 10.1002/mdc3.13941, PMID: 38386479 PMC10883406

[29] Bond AE Shah BB Huss DS Dallapiazza RF Warren A Harrison MB. Safety and efficacy of focused ultrasound Thalamotomy for patients with medication-refractory, tremor-dominant Parkinson disease: a randomized clinical trial. JAMA Neurol. (2017) 74:1412–8. doi: 10.1001/jamaneurol.2017.3098, PMID: 29084313 PMC5822192

[30] Elias WJ Lipsman N Ondo WG Ghanouni P Kim YG Lee W. A randomized trial of focused ultrasound Thalamotomy for essential tremor. N Engl J Med. (2016) 375:730–9. doi: 10.1056/NEJMoa1600159, PMID: 27557301

[31] Ghanouni P Pauly KB Elias WJ Henderson J Sheehan J Monteith S. Transcranial MRI-guided focused ultrasound: a review of the technologic and neurologic applications. AJR Am J Roentgenol. (2015) 205:150–9. doi: 10.2214/AJR.14.13632, PMID: 26102394 PMC4687492

[32] Park Y-S Jung NY Na YC Chang JW. Four-year follow-up results of magnetic resonance-guided focused ultrasound thalamotomy for essential tremor. Mov Disord. (2019) 34:727–34. doi: 10.1002/mds.27637, PMID: 30759322

[33] Purrer V Pohl E Borger V Weiland H Boecker H Schmeel FC. Motor and non-motor outcome in tremor dominant Parkinson's disease after MR-guided focused ultrasound thalamotomy. J Neurol. (2024) 271:3731–42. doi: 10.1007/s00415-024-12469-z, PMID: 38822147 PMC11233288

[34] Gauthier-Lafreniere E Aljassar M Rymar VV Milton J Sadikot AF. A standardized accelerometry method for characterizing tremor: application and validation in an ageing population with postural and action tremor. Front Neuroinform. (2022) 16:878279. doi: 10.3389/fninf.2022.878279, PMID: 35991289 PMC9386269

[35] Herzog J Hamel W Wenzelburger R Pötter M Pinsker MO Bartussek J. Kinematic analysis of thalamic versus subthalamic neurostimulation in postural and intention tremor. Brain. (2007) 130:1608–25. doi: 10.1093/brain/awm077, PMID: 17439979

[36] Papapetropoulos S Gallo BV Guevara A Singer C Mitsi G Lyssikatos C. Objective tremor registration during DBS surgery for essential tremor. Clin Neurol Neurosurg. (2009) 111:376–9. doi: 10.1016/j.clineuro.2008.10.017, PMID: 19121890

[37] Paschen S Forstenpointner J Becktepe J Heinzel S Hellriegel H Witt K. Long-term efficacy of deep brain stimulation for essential tremor: an observer-blinded study. Neurology. (2019) 92:e1378–86. doi: 10.1212/WNL.0000000000007134, PMID: 30787161

[38] Wastensson G Holmberg B Johnels B Barregard L. Quantitative methods for evaluating the efficacy of thalamic deep brain stimulation in patients with essential tremor. Tremor Other Hyperkinet Mov. (2013) 3:tre-03-196-4279-2. doi: 10.7916/D8VM4B0N, PMID: 24255800 PMC3822146

[39] Swytink-Binnema CA Coreas A Pichardo S Pike GB Kiss ZH. An intraoperative accelerometry and real-time analysis tool for magnetic resonance-guided focused ultrasound thalamotomy. J Neurosurg. (2024) 141:1088–95. doi: 10.3171/2024.1.JNS231830, PMID: 38626471

[40] Baek H Chen J Lockwood D Obusez E Poturalski M Nagel SJ. Feasibility of magnetic resonance-compatible accelerometers to monitor tremor fluctuations during magnetic resonance-guided focused ultrasound Thalamotomy: technical note. Oper Neurosurg. (2023) 24:641–50. doi: 10.1227/ons.0000000000000638, PMID: 36827201

