# Supplementary material for: Quantitative and qualitative tremor evaluation after MR-guided focused ultrasound thalamotomy
Source: Front Neurol. 2025 May 2;16:1594382. doi: 10.3389/fneur.2025.1594382 (PMC12081243; doi:10.3389/fneur.2025.1594382)
Supplement: Supplementary file 1 [file Data_Sheet_1.docx]

Supplementary Material

**Figure S1**. Study design

**Methods S1.** Subscore calculation

**Methods S2.** Accelerometry analysis

**Figure S2.** Graphical user interface of the proprietary software

**Figure S3.** Visualization of the data after manual preprocessing

**Figure S4.** Resulting raw data after filtering and coding

**Figure S5.** Normalized power spectrum

**Figure S6.** Example of individual power spectra in an ET and PD patient

**Figure S7.** Example of an individual power spectra before and after MRI-guided focused ultrasound treatment

**Figure S8.** Changes in tremor characteristics after MRI-guided focused ultrasound thalamotomy

Table S1. Qualitative and quantitative tremor outcome after MRgFUS


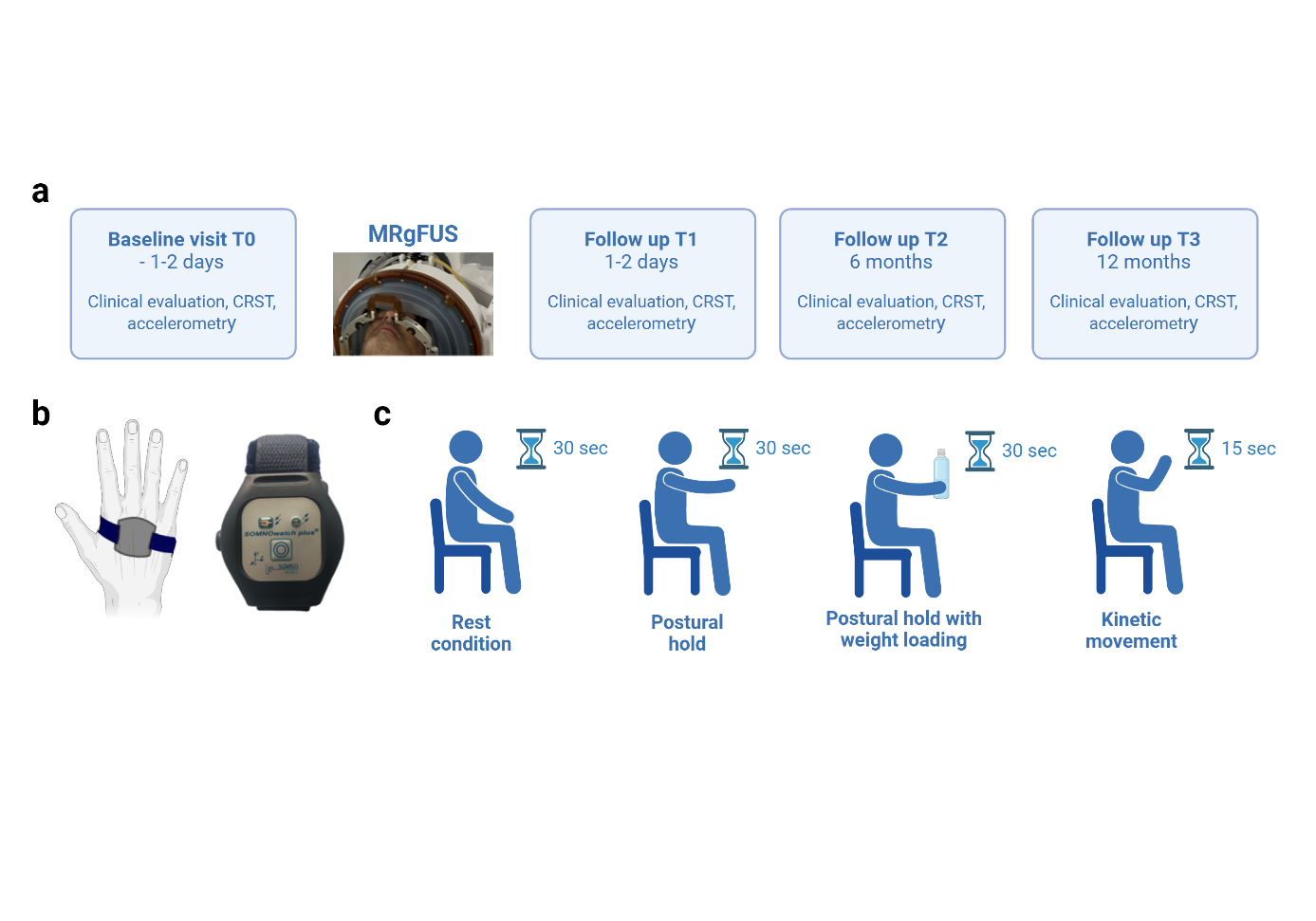


**Suppl. Fig. S1 Study design.** (a) The study included a baseline visit prior to treatment and three follow up visits within 12 months. At each point a clinical evaluation as well as tremor evaluation using the Clinical Rating Scale for Tremor (CRST) and triaxial accelerometry were conducted. (b) The triaxial accelerometer was placed on the proximal one-third of the metacarpus. (c) Tremor recordings were obtained bilaterally while rest, forward outstretched postural condition (without and with weight loading) and kinetic (finger-to-nose maneuver) condition.

**Supplementary methods S1.** Subscore calculation

Clinical Rating Scale for Tremor:

The modified score of the treated upper extremity was derived from the CRST, part A (3 items, rest, postural and kinetic condition of the treated upper extremity) and part B (4 items, drawing A-C and pouring of the treated side). Each item ranged from 0 to 4; the total score ranged from 0 to 28. Higher values indicated more severe symptoms.

The modified score of the untreated upper extremity was derived from the CRST, part A (3 items, rest, postural and kinetic condition of the untreated upper extremity) and part B (4 items, drawing A-C and pouring of the untreated side). Each item ranged from 0 to 4; the total score ranged from 0 to 28. Higher values indicated more severe symptoms.

**Supplementary methods S2.** Accelerometry analysis

Data acquisition:

Tremor recordings were preprocessed using the DOMINOlight software (SOMNOmedics, Randersacker, Germany). During recordings, the different exercises are separated by “event markers” (labelled with the German word “Patientenmarker” = patient marker), which are displayed in the application view (Suppl. Fig 1). The first and last 5-10 seconds of each recording (depending on the clinical observation recorded during the measurement) were removed to avoid measurements of arbitrary movements for initiating or terminating the exercise or distortions of the power spectrum caused by the short-term arrest in re-emergent tremor and the new markers were named accordingly to the exercise condition (e.g. “Start rest condition right”). Then, the sections between start and end points were defined (corresponding to the different coloring in Suppl. Fig. 2) and downloaded separately. Accelerometer data of each tremor condition were downloaded in raw.txt format.

Further analysis was carried out using Matlab (MathWorks, Inc., USA, R2023b).


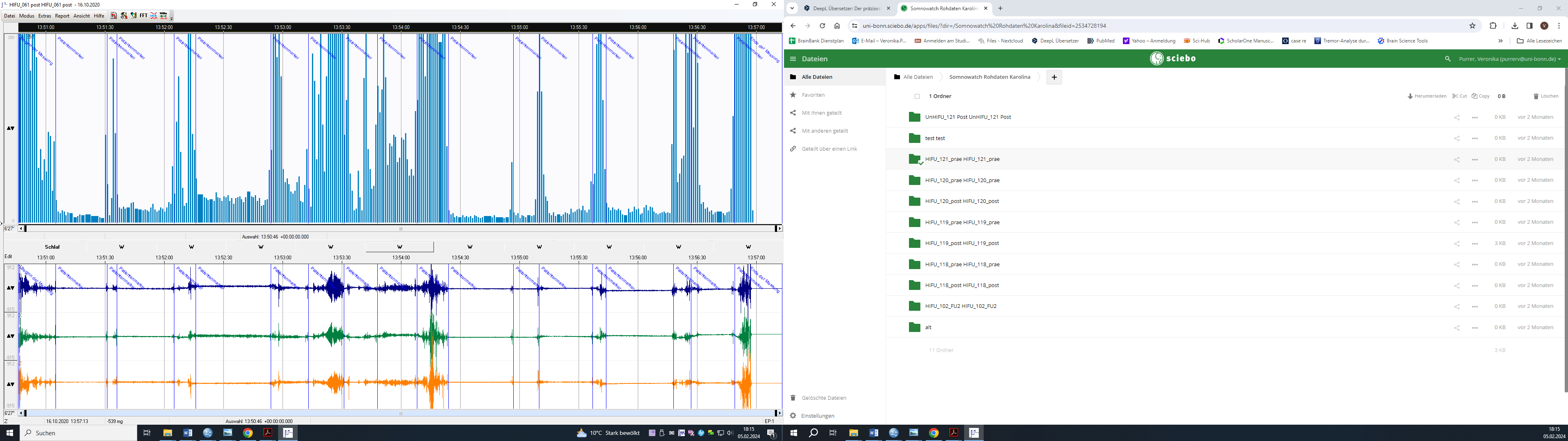


**Suppl. Fig. S2: Graphical user interface of the proprietary software** (DOMINOlight; SOMNOmedics visualizing the timeline of accelerometric recordings. The different exercises are separated by “event markers”, indicating the start and end of the condition (labelled with the German word “Patientenmarker” = patient marker). The upper row displays the activity (in mg). The lower row shows the raw data from the acquisition for the x- (green), y- (blue) and z-axis (orange).


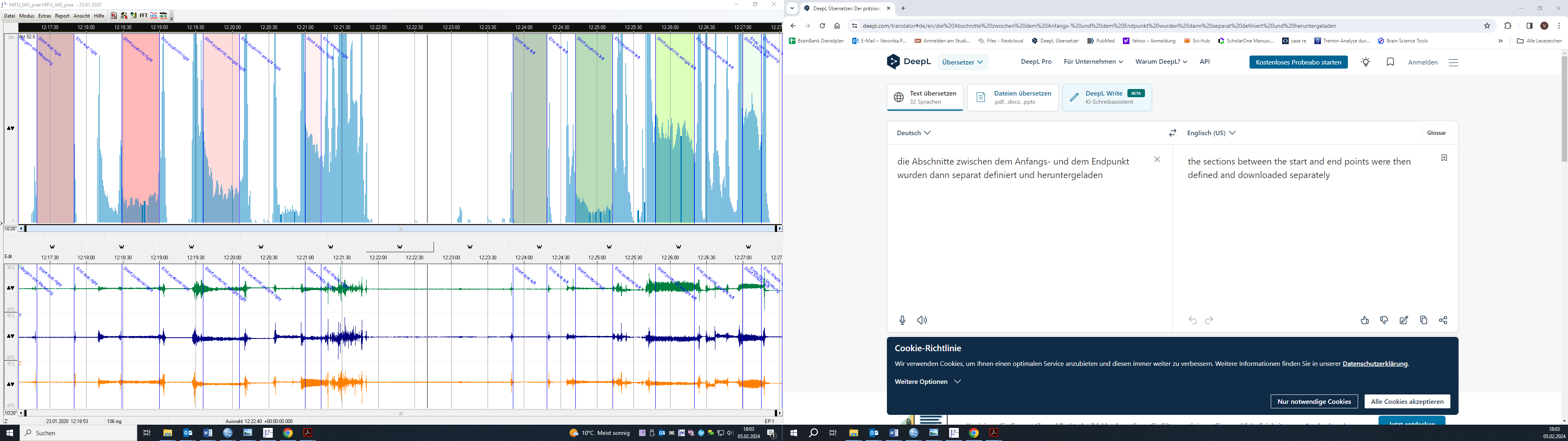


**Suppl. Fig. S3: Visualization of the data after manual preprocessing:** removal of arbitrary movements or distortions of the power spectrum, relabeling of the event markers according to the exercise condition, definition of the time interval from the start to the end point of each condition.

Data filtering and coding:

The raw data is comprised of 3 vectors: x, y, and z accelerations (units are recorded in mg). A bandpass filter from 1 to 20 Hz with a finite impulse response filter of the 250^th^ order and linear detrending (Matlab function *filtfilt* and *detrend*) was used to remove voluntary movements (low frequency) or random noise (high frequency) but maintain a wide bandwidth covering all known tremor frequencies. Using a principal component analysis (Matlab function *pca*), the three accelerometric sources (x,y,z) were divided into three principal components and arranged in descending order of component variance (Suppl. Fig 3). As mentioned above, the first principal component comprises the largest possible variance and therefore most likely contains the main direction of tremor oscillation. This component was used to generate the power spectrum.


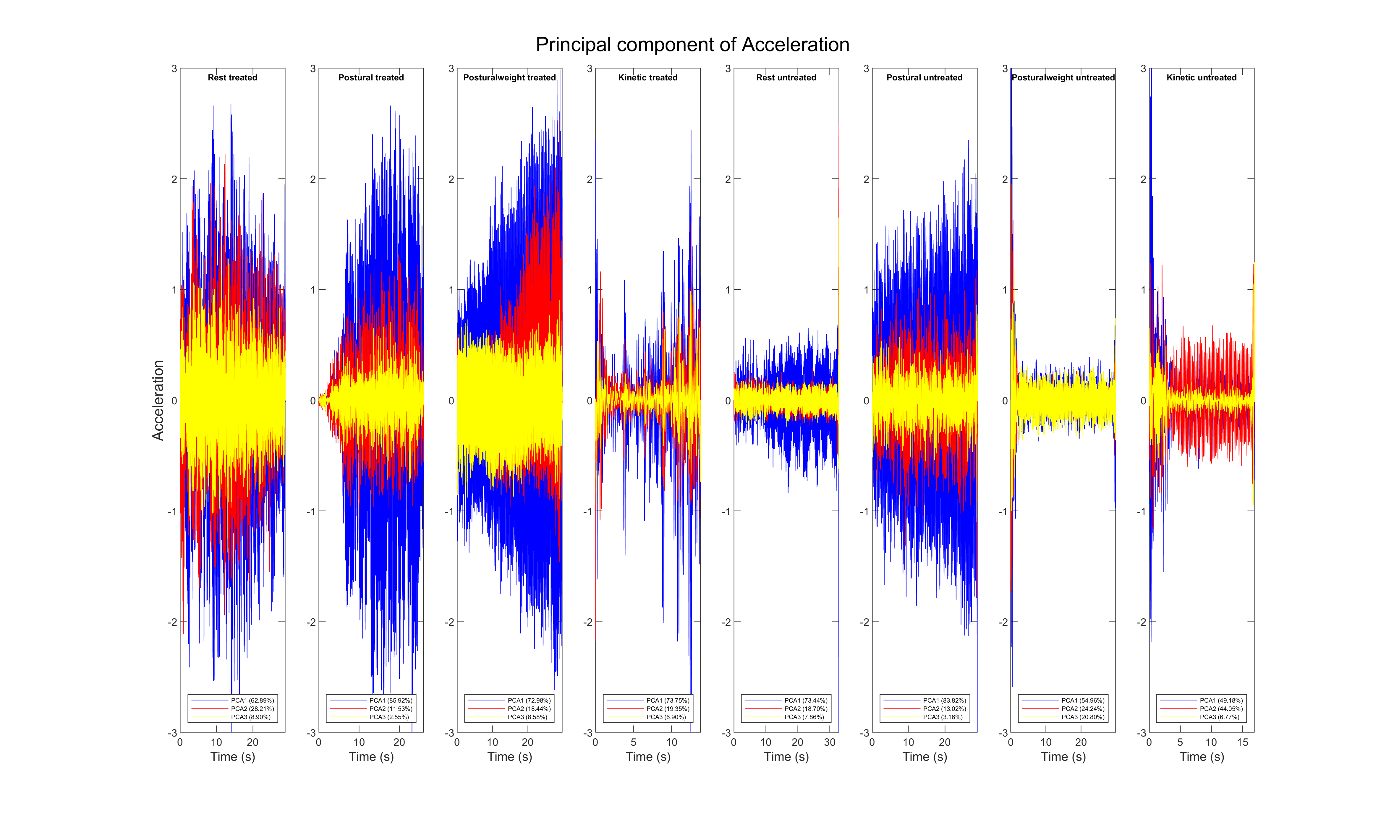


**Suppl. Fig. S4: Resulting raw data after filtering and coding.** A full recording of both arms of a patient with PD is presented. The blue, red and yellow traces represent the three principal components, arranged in descending order of component variance. The first principal component was used for further analysis.

Data analysis:

The power spectrum of the first principal component of the signal was computed using a hamming window and a Fast Fourier Transform (Matlab function *hamming* and *fft*). Subsequently, the following spectral parameters were extracted:

- Peak frequency (f_p_) between 2-15 Hz (in Hz) = frequency with the maximum power in the power spectrum within the range of 2-15 Hz
- Frequency Width at Half Maximum (FWHM) (in Hz) = frequency bandwidth where the power spectrum's value is at least half of its maximum value. The FWHM is a measure of the range of different frequencies within the entire signal better characterize the shifting between multiple oscillators ^1^
- Tremor stability index (TSI) (in Hz) = interquartile range of the change in frequency (Δf). This cycle-by-cycle variations in tremor frequency were calculated from corresponding series of instantaneous frequencies (*f_n_*) using the following function: Δf = fn − fn+1. This metric measures the short-term stability or temporal aspect of tremor and was established to differentiate tremors in ET patients from PD patients ^2^
- Half-width power (HWP) (in milligravities (mg)) = power referring to the frequency bandwidth where the power spectrum maintains at least half of its maximum value ^1^

***
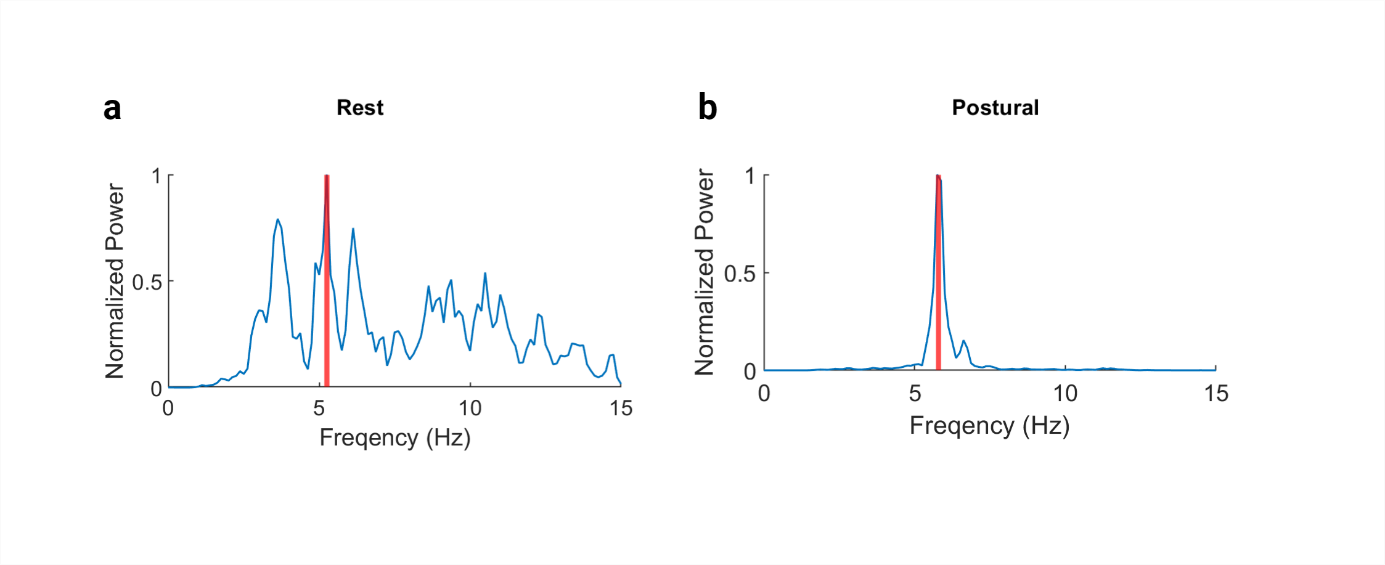
***

**Suppl. Fig. S5: Normalized power spectrum** of a patient with essential tremor with an obvious tremor peak during posture (a) and no obvious peak at rest (b). The red vertical line marks the peak frequency.

*
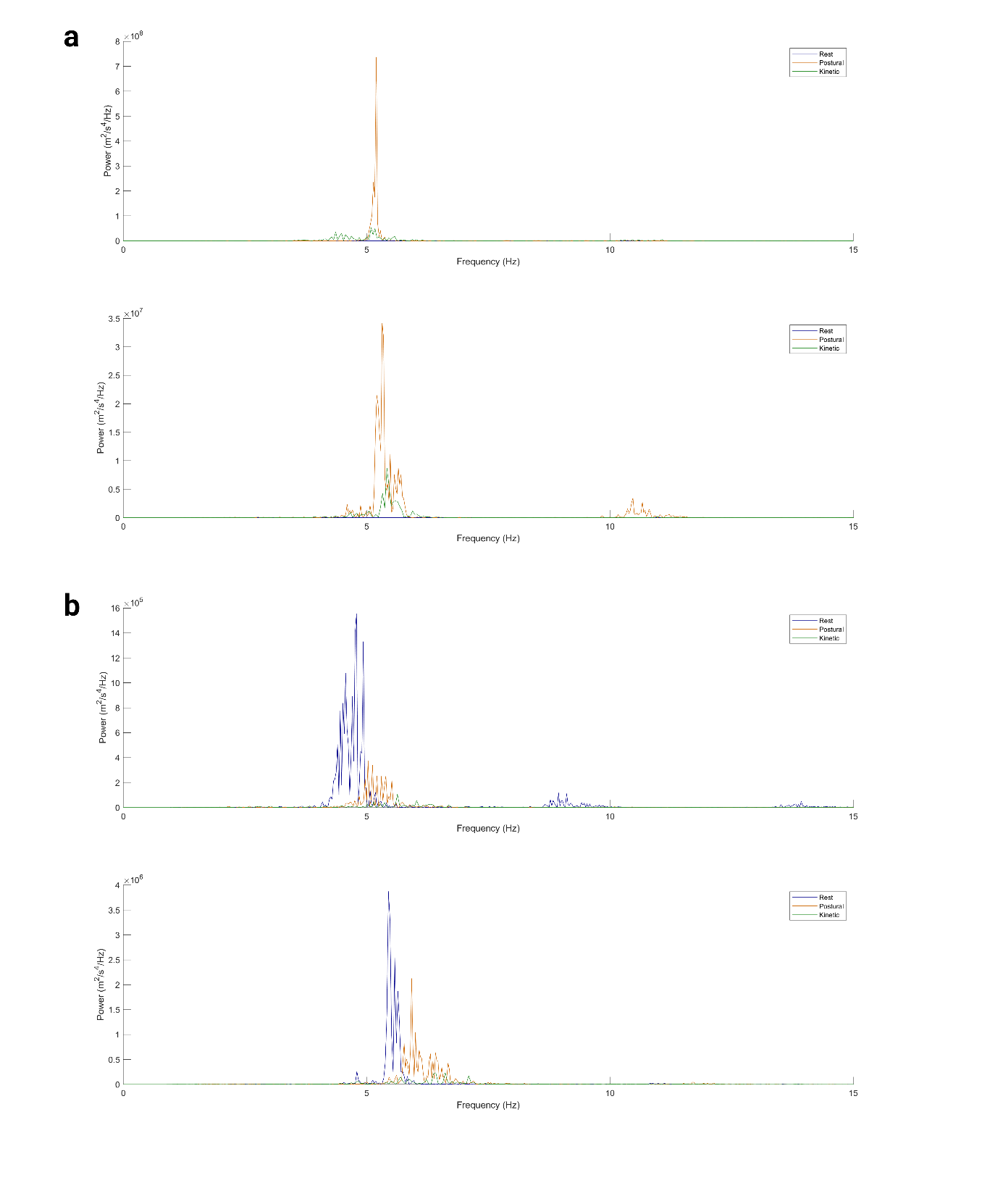
*

**Suppl. Fig. S6: Example of individual power spectra in an ET and PD patient.** The combined power spectra for the three conditions (rest (*blue*), posture (*orange*) and kinetic (*green*)) are shown for the treated and untreated extremity separately. (a) In ET, HWP was highest at postural condition, while rest condition showed the highest power in PD. Peak frequencies remained stable over all conditions.

*
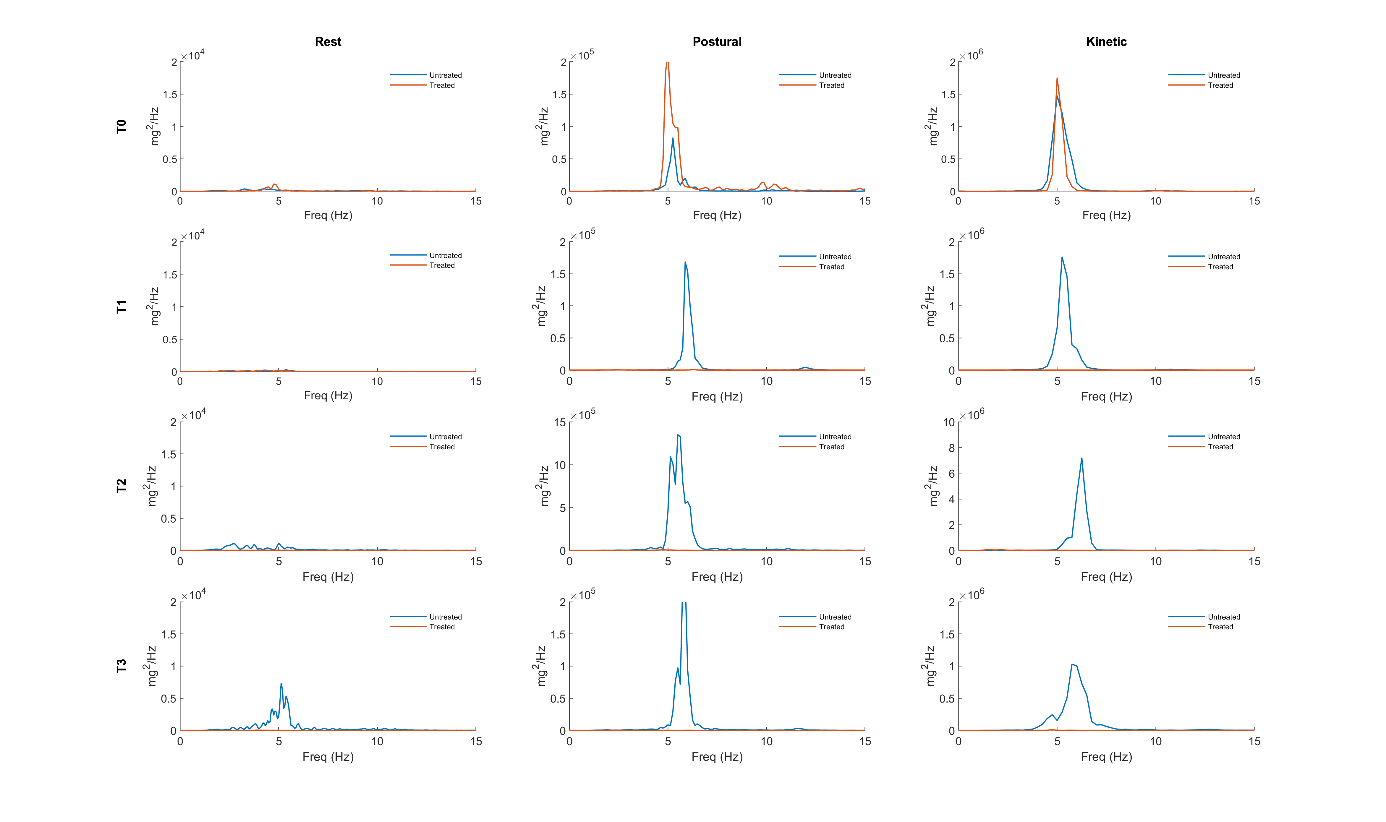
***Suppl. Fig. S7: Example of an individual power spectra before and after MRI-guided focused ultrasound treatment.** Power spectra are provided for each condition and time point separately. A significant tremor reduction was observed in the treated (*orange*) extremity at all time points of follow-up.


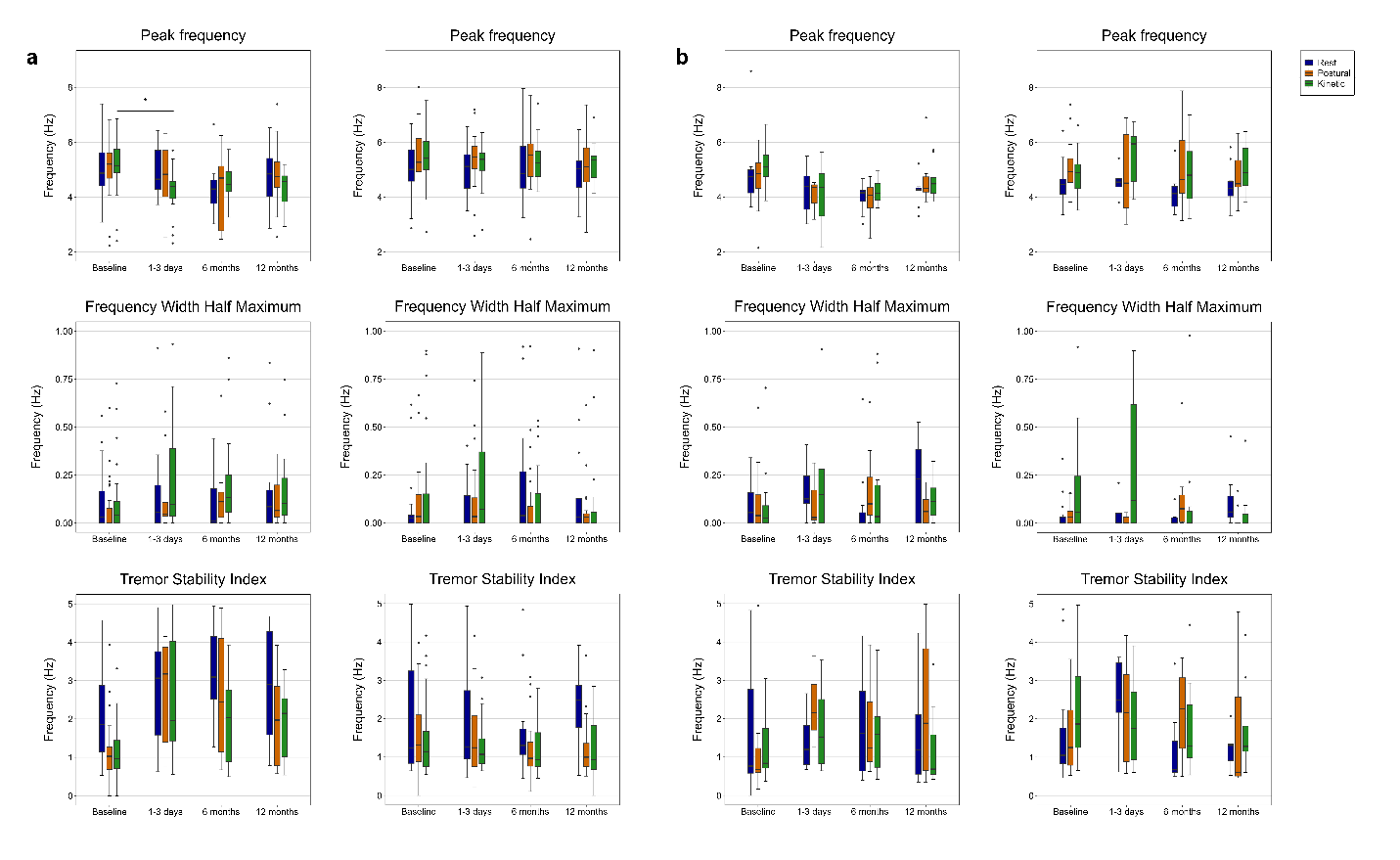


**Suppl. Fig. S8. Changes in tremor characteristics after MRI-guided focused ultrasound thalamotomy** in patients with (a) essential tremor (ET) and (b) Parkinson’s disease (PD). Boxplots of peak frequency (f_p_), tremor stability index (TSI) and full-width half maximum (FWHM) are shown for the treated (*first column*) and untreated (*second column*) extremity separately.

**Suppl. Tbl. S1 Qualitative and quantitative tremor outcome after MRgFUS**

| **ET** | **Timepoint** | | | | |
| --- | --- | --- | --- | --- | --- |
|  | **T0 (n=35)** | **Friedman test value*** | **T1 (n=35)**^#^ | **T2 (n=35)**^#^ | **T3 (n=30)**^#^ |
| CRST  Total score | 59.8 ± 17.1 | χ2(3)=57.43  p<0.001 | 28.9 ± 10.1  (r=1.19, p<0.001) | 30.9 ± 13.6  (r=1.15, p<0.001) | 31.2 ± 14.9  (r=0.93, p<0.001) |
| (CRST_mod_)^‡^  Treated arm | 19.1 ± 4.9 | χ2(3)=62.52  p<0.001 | 4.3 ± 3.0  (r=1.27, p<0.001) | 6.5 ± 5.0  (r=0.98, p<0.001) | 6.3 ± 4.5  (r=0.80, p<0.001) |
| (CRST_mod_)^‡^  Untreated arm | 16.9 ± 5.6 | χ2(3)=4.23  p=0.237 | 16.4 ± 5.6 | 16.4 ± 6.3 | 6.1 ± 5.1 |
| (CRST_R_)^‡^  Treated arm | 0.7 ± 0.9 | χ2(3)=37.53  p<0.001 | 0.0 ± 0.0  (r=0.47, p=0.017) | 0.0 ± 0.0  (r=0.47, p=0.017) | 0.0 ± 0.2  (r=0.43, p=0.020) |
| (CRST_R_)^‡^  Untreated arm | 0.5 ± 0.7 | χ2(3)=1.28  p=0.734 | 0.5 ± 0.7 | 0.6 ± 0.9 | 0.6 ± 0.8 |
| (CRST_P_)^‡^  Treated arm | 3.1 ± 0.8 | χ2(3)=63.53  p<0.001 | 0.5 ± 0.7  (r=1.15, p<0.001) | 0.6 ± 0.6  (r=1.04, p<0.001) | 0.7 ± 0.7  (r=0.93, p<0.001) |
| (CRST_P_)^‡^  Untreated arm | 2.7 ± 0.9 | χ2(3)=3.83  p=0.280 | 2.4 ± 1.0 | 2.5 ± 1.0 | 2.6 ± 0.9 |
| (CRST_K_)^‡^  Treated arm | 3.2 ± 0.9 | χ2(3)=67.35  p<0.001 | 0.5 ± 0.6  (r=1.34, p<0.001) | 0.9 ± 0.9  (r=0.98, p<0.001) | 1.1 ± 1.0  (r=0.82, p<0.001) |
| (CRST_K_)^‡^  Untreated arm | 2.8 ± 1.1 | χ2(3)=0.77  p=0.857 | 2.7 ± 1.3 | 2.8 ± 1.1 | 3.0 ± 1.2 |
| (HWP_R_)^‡^  Treated arm | 864.1 ± 1887.1 | χ2(3)=7.61  p=0.055 | 65.7 ± 123.7 | 80.9 ± 287.5 | 74.6 ± 113.8 |
| (HWP_R_)^‡^  Untreated arm | 583.6 ± 1727.5 | χ2(3)=2.15  p=0.54 | 3170.2 ± 10980.9 | 883.8 ± 4309.4 | 958.6 ± 2041.6 |
| (HWP_P_)^‡^  Treated side | 23315.0 ± 32370.6 | χ2(3)=35.05  p<0.001 | 2204.9 ± 6484.8  (r=0.71, p<0.001) | 926.2 ± 1876.9  (r=0.75, p<0.001) | 600.0 ± 1315.1  (r=0.75, p<0.001) |
| (HWP_P_)^‡^  Untreated side | 18653.4 ± 36314.4 | χ2(3)=1.75  p=0.63 | 21737.3 ± 34864.4 | 13864.1 ± 29958.5 | 52036.1 ± 220430.2 |
| (HWP_K_)^‡^  Treated side | 162348.3± 435421.5 | χ2(3)=27.63  p<0.001 | 2343.2 ± 8762.0  (r=0.78, p<0.001) | 3266.7 ± 10200.4  (r=0.58, p<0.001) | 940.7 ± 1717.7  (r=0.56, p<0.001) |
| (HWP_K_)^‡^  Untreated side | 127298.0 ± 289200.6 | χ2(3)=3.18  p=0.36 | 239035.9 ± 552122.5 | 116442.3 ± 238964.0 | 163050.5 ± 274545.6 |
| **PD** | **Timepoint** | | | | |
|  | **T0 (n=21)** | **Friedman test value*** | **T1 (n=21)** ^#^ | **T2 (n=21)** ^#^ | **T3 (n=19)** ^#^ |
| CRST  Total score | 31.0 ± 15.0 | χ2(3)=32.19  p<0.001 | 11.7 ± 9.4  (r=1.25, p<0.001) | 17.0 ± 7.9  (r=0.60, p=0.012) | 15.1 ± 7.5  (r=1.00, p<0.001) |
| (CRST_mod_)^‡^  Treated arm | 13.8 ± 5.5 | χ2(3)=38.23  p<0.001 | 2.5 ± 1.8  (r=1.46, p<0.001) | 5.2 ± 2.8  (r=0.75, p=0.001) | 4.9 ± 2.9  (r=0.87, p<0.001) |
| (CRST_mod_)^‡^  Untreated arm | 6.0 ± 5.3 | χ2(3)=1.44  p=0.697 | 5.8 ± 5.5 | 5.9 ± 4.1 | 6.0 ± 5.1 |
| (CRST_R_)^†^  Treated arm | 3.4 ± 0.5 | χ2(3)=40.68  p<0.001 | 0.1 ± 0.4  (r=1.37, p<0.001) | 1.3 ± 1.2  (r=0.72, p=0.002) | 1.1 ± 1.1  (r=0.84, p<0.001) |
| (CRST_R_)^†^  Untreated arm | 0.9 ± 0.9 | χ2(3)=4.83  p=0.185 | 0.8 ± 1.0 | 1.1 ± 1.3 | 0.8 ± 1.1 |
| (CRST_P_)^†^  Treated arm | 2.7 ± 1.1 | χ2(3)=34.49  p<0.001 | 0.2 ± 0.4  (r=1.21, p<0.001) | 0.9 ± 0.9  (r=0.63, p=0.008) | 0.8 ± 0.8  (r=0.69, p=0.005) |
| (CRST_P_)^†^  Untreated arm | 1.0 ± 1.1 | χ2(3)=3.27  p=0.352 | 0.8 ± 0.8 | 0.7 ± 0.8 | 0.8 ± 0.8 |
| (CRST_K_)^†^  Treated arm | 1.4 ± 1.2 | χ2(3)=23.46  p<0.001 | 0.2 ± 0.4  (r=0.78, p=0.002) | 0.4 ± 0.5  (r=0.59, p=0.018) | 0.4 ± 0.5  (r=0.69, p=0.005) |
| (CRST_K_)^†^  Untreated arm | 0.6 ± 0.9 | χ2(3)=4.25  p=0.236 | 0.7 ± 0.8 | 0.4 ± 0.6 | 0.6 ± 0.8 |
| (HWP_R_)  Treated arm | 4132.6 ± 6683.6 | χ2(3)=9.55  p=0.023 | 113.8 ± 299.1  (p=0.324) | 1440.4 ± 4806.8 (r=0.53, p=0.045) | 2368.8 ± 8543.7 (r=0.53, p=0.045) |
| (HWP_R_)  Untreated arm | 387.7 ± 895.1 | χ2(3)=1.03  p=0.795 | 264.2 ± 948.2 | 912.0 ± 3268.4 | 2867.4 ± 7481.9 |
| (HWP_P_)  Treated side | 53153.8 ± 114020.9 | χ2(3)=20.79  p<0.001 | 199.5 ± 698.9  (r=0.69, p=0.005) | 107.0 ± 228.7 (r=0.74, p=0.004) | 910.4 ± 2444.0 (r=0.64, p=0.007) |
| (HWP_P_)  Untreated side | 3834.2 ± 8372.1 | χ2(3)=3.86  p=0.277 | 2048.0 ± 4046.8 | 1733.2 ± 3663.1 | 2193.6 ± 3374.5 |
| (HWP_K_)  Treated side | 37923.2 ± 65399.4 | χ2(3)=12.75  p=0.005 | 806.5 ± 2765.0 (r=0.64, p=0.020) | 3449.7 ± 14196.8 (r=0.51, p=0.041) | 2047.0 ± 6583.8 (r=0.51, p=0.041) |
| (HWP_K_)  Untreated side | 2190.2 ± 3565.9 | χ2(3)=4.15  p=0.246 | 4288.4 ± 9491.0 | 5860.1 ± 14067.2 | 1657.2 ± 2644.3 |
| Values are means ± SD.  * P values are based on the Friedman Test (p<0.05). Patients with completed 12-months follow-up were included.  ^#^ Pairwise post hoc analysis was conducted for all follow-up time points compared to baseline and false discovery rate (FDR) corrections for multiple comparisons were applied.  ^‡^ The modified score was derived from the CRST, part A (3 items) and part B (4 items) for the treated and untreated upper extremity (range 0 to 28).  ^†^ Item for rest, postural or kinetic tremor in the treated or untreated upper limb (Part A); each item ranges from 0 to 4.  Abbreviations: MRgFUS = Magnetic Resonance-guided Focused Ultrasound; T0 = Baseline; T1 = 1-3 days post-MRgFUS; T2 = 6 months post-MRgFUS; T3 = 12 months post-MRgFUS; CRST = Clinical Rating Scale for Tremor; CRST_mod_ = modified score of the Clinical Rating Scale for Tremor; CRST_R_ = subitem for rest tremor; CRST_P_ = subitem for postural tremor; CRST_K_ = subitem for kinetic tremor; HWP_R_ = half-width power while rest; HWP_P_ = half-width power while posture; HWP_K_ = half-width power while kinetic movement | | | | | |

**References**

1. Panyakaew P, Cho HJ, Lee SW, Wu T, Hallett M. The Pathophysiology of Dystonic Tremors and Comparison With Essential Tremor. *J Neurosci*. 2020;40(48):9317-9326. doi:10.1523/JNEUROSCI.1181-20.2020

2. Di Biase L, Brittain J-S, Shah SA, et al. Tremor stability index: a new tool for differential diagnosis in tremor syndromes. *Brain*. 2017;140(7):1977-1986. doi:10.1093/brain/awx104
